# Size Variation under Domestication: Conservatism in the inner ear shape of wolves, dogs and dingoes

**DOI:** 10.1038/s41598-017-13523-9

**Published:** 2017-10-17

**Authors:** Anita V. Schweizer, Renaud Lebrun, Laura A. B. Wilson, Loïc Costeur, Thomas Schmelzle, Marcelo R. Sánchez-Villagra

**Affiliations:** 1 Paläontologisches Institut und Museum, Karl-Schmid-Strasse 4, 8006 Zürich, Switzerland; 2 Laboratoire de Paléontologie, Institut des Sciences de l’Evolution de Montpellier, UMR-CNRS 5554, cc 064, Université de Montpellier 2, 34095 Montpellier Cedex 5, France; 3 PANGEA Research Centre, School of Biological, Earth and Environmental Sciences, University of New South Wales, Sydney, NSW 2052 Australia; 4 Naturhistorisches Museum Basel, Augustinergasse 2, 4051 Basel, Switzerland; 5 Fleckensteinstraße 7, 74206 Bad Wimpfen, Germany

## Abstract

A broad sample of wolves, dingoes, and domesticated dogs of different kinds and time periods was used to identify changes in size and shape of the organs of balance and hearing related to domestication and to evaluate the potential utility of uncovered patterns as markers of domestication. Using geometric morphometrics coupled with non-invasive imaging and three-dimensional reconstructions, we exposed and compared complex structures that remain largely conserved. There is no statistically significant difference in the levels of shape variation between prehistoric and modern dogs. Shape variance is slightly higher for the different components of the inner ear in modern dogs than in wolves, but these differences are not significant. Wolves express a significantly greater level of variance in the angle between the lateral and the posterior canal than domestic dog breeds. Wolves have smaller levels of size variation than dogs. In terms of the shape of the semicircular canals, dingoes reflect the mean shape in the context of variation in the sample. This mirrors the condition of feral forms in other organs, in which there is an incomplete return to the characteristics of the ancestor. In general, morphological diversity or disparity in the inner ear is generated by scaling.

## Introduction

The tremendous morphological disparity in dogs as compared to its ancestral species, the wolf, is well-recognized, with previous quantitative studies having concentrated on skull shape^1–5^ and life history variables^6^. We examine here a feature of the skull that is of relevance for understanding cranio-sensory changes related to domestication: the inner ear.

The inner ear consists of the organs of balance and hearing, with an intricate shape as revealed by the bony labyrinth^7^ in the internal structures of the petrosal bone^8,9^. Advances in tomographic techniques and methods to quantify three-dimensional (3D) surfaces make it possible to expose and visualize the petrosal internally, and to analyse the shape of the semicircular canals and of the cochlea^10–13^. The petrosal is one of the most dense and taxonomically informative bones of the mammalian skeleton, and is often used in studies of ancient DNA^14^. However, it is usually neglected in zooarchaeological studies, as anatomical studies have not been conducted for comparisons of domesticated versus wild forms, now an easier task given the possibility of non-invasive imaging of this complex structure.

Some differences between dogs and wolves have been found in some cranio-sensory areas. Many parameters of the ear ossicles are diverse among breeds of dogs and different to those recorded in wolves^15,16^. Domestication in canids is known to have modified the genetic determinants of senses such as olfaction, albeit not to a significant level and not to the level that it changed the olfactory capacities of domesticated dogs that were also probably selected for them^17^. In contrast, a clear decrease in brain size resulting from dog domestication has been quantified and discerned in its anatomical details^3,18^. All in all, these works show that physical changes in sensory parts of the skull can occur during domestication, but detecting and quantifying them requires a clear understanding of intraspecific variation.

The interspecific variation of the inner ear is well-documented for several mammalian clades^10,12,19,20^. Although the general structure of the semicircular canals and the cochlea is highly conserved, the proportions among structures and the number of cochlear coils are variable features, some of which are correlated with locomotory habits and hearing frequencies^21–29^. Much less is known about intraspecific variation in the inner ear, although recent works have started to document this^12,20,30^. The limited amount of information at hand indicates a relatively small morphological variability of the structure within species^20^, making it a good taxonomic marker. Dogs, wolves and dingoes belong to the same species. One question is to understand if domestication or return to wildlife in the case of the dingo, has led to more morphological variability in the inner ear, or even to a return to a putative ancestral state^31^. Some studies conducted on natural populations suggest that a decreased functional importance of parts of the vestibular organ led to more variation within a species of sloth^19,32^, recalling relaxation of selective constraints leading to high levels of variation observed in the genome of dogs when compared to wolves^33^. Likewise, an experimental study on laboratory mice concluded that the shape of the semicircular canals was affected by selective breeding for increased locomotion even over a small number of generations, whereas canal size remained unchanged^34^.

The aim of this study is to quantify and compare the shape of the organs of balance and hearing in wolves versus dogs and dingoes. To explore variation in morphology associated with domestication, we examine specimens that represent both the first phases of domestication as well as intensive selective breeding, in addition to the dingo, a special case of a wild canid closely resembling domestic dogs. We use 3D geometric morphometric methods to quantify variation in the bony markers of the complex structures involved. This approach provides visually-intuitive insights into the cranio-sensory changes related to domestication; it also serves to test for potential new osteological markers of domestication with tools that can discern subtle changes in shape and the effects of size.

## Methods

**Figure 1:**
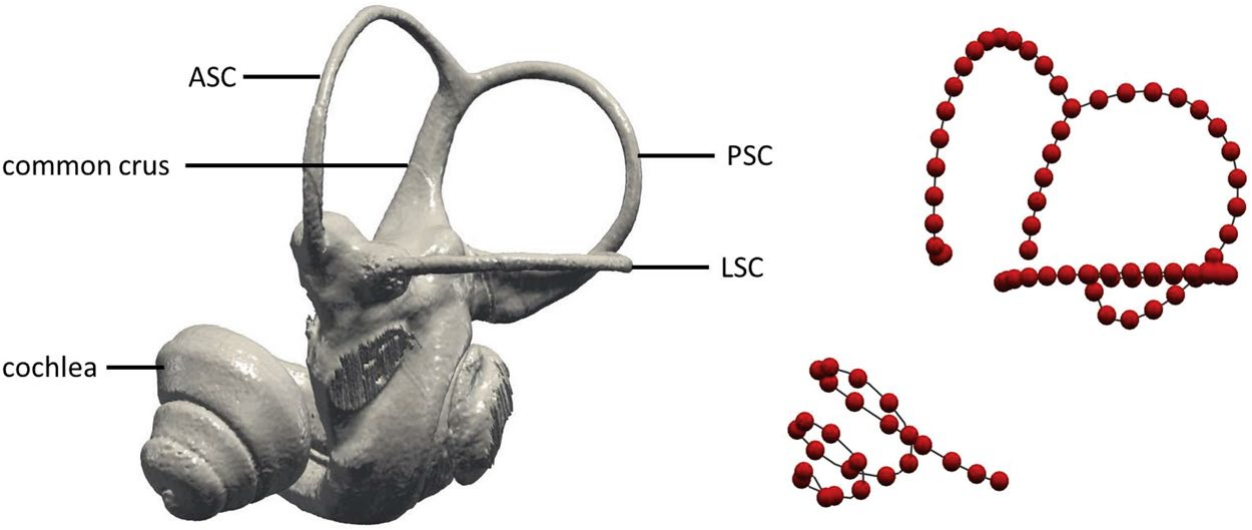
3D model of a bony labyrinth and semilandmark locations. Left: three-dimensional virtual endocast of the left inner ear of a wolf (ZMUZH 17603) in lateral view (LSC: lateral semicircular canal; ASC: anterior semicircular; PSC: posterior semicircular canal). Right: location of the semilandmarks along the structures of interest after resampling and sliding.

We sampled 92 canid skulls (Supplementary Table 1), comprising 39 modern dogs (*Canis lupus familiaris*) from 20 different breeds, 21 prehistoric specimens, 24 wolves (*Canis lupus*) as well as eight dingoes (*Canis lupus dingo*), constituting four groups we analyse accordingly. Dingoes are considered by many as wild canids, while others emphasize the uniqueness of this form in more formal taxonomic nomenclature^35^. The sample includes adults and subadults (Supplementary Table 1). This does not influence the results since the bony labyrinth is already ossified prenatally in placentals^36^. The prehistoric samples are from various finding sites, which were used to estimate the age of the skulls (Supplementary Table 2). The skulls were scanned using high-resolution x-ray micro-computed tomography (CT) at different facilities (Supplementary Tables 3 and 4). 3D surface models of the left inner ear of the specimens were generated in Avizo® 8.0, Avizo® 8.1.0. (FEI Visualization Science Group, Germany) and in Geomagic Wrap® 2013 64-Bit-Version (2013.0.1.1206). If the left inner ear was incomplete, the right inner ear was modelled and mirrored. After segmentation, the generated surfaces were extracted from Avizo® as smoothed 3D surfaces (STL files) for further analyses (Fig. 1). As a proxy for size, the cranial base length^37^ was measured when as possible (Supplementary Table 1).

There were no experiments conducted in any living animal in this study, and all the specimens used were skulls deposited in museums and academic institutions – thus, all methods were conducted in accordance to relevant guidelines and regulations.

We used a semilandmark approach to quantify the complex morphology of the inner ear. The 3D STL surface files were imported into the software ISE-MeshTools 1.0.4^38^ in order to digitize 3D curves along the semicircular canals and the cochlea (Fig. 1). The starting and ending points of the curves were at fixed locations (Supplementary Table 5), which corresponds to Type I landmarks^39^. In a second step, these curves were re-expressed as equidistant semilandmarks^40^. In order to give an equal weight to the four analysed structures (the three semicircular canals and the cochlea) in the subsequent analyses, we retained a number of 25 equidistant semilandmarks for the cochlea, 25 for the lateral semicircular canal, 18 for the anterior and the posterior semicircular canal and seven for the common crus. Since the common crus is shared by the anterior and the posterior semicircular canals, this adds to a total of 25 semilandmarks for the anterior and the posterior canal.

Prior to multivariate analyses, the semilandmarks were slid along the curves using the minimized Procrustes distance criterion to discard information that would result from the arbitrary initial equidistant spacing of the semilandmarks along the curves, in order to increase geometric equivalence^21,32,39–41^. A generalized procrustes superimposition was performed on the slid semilandmarks using the interactive software package MorphoTools^42–44^ to remove the effects of scale, orientation and position.

We used principal component analysis (PCA) in order to visualize independent patterns of variation that are contained within the semilandmark data^45^. A shape regression of PC2 against centroid size was plotted using the software morphoJ 1.06d^46^.

In order to study the length of the common crus, and other aspects of the orientation and configuration of the labyrinth and semicircular canals, we conducted additional analyses. As the length of the common crus may vary within the four investigated dog groups, we measured the length of the common crus (CCL) for all specimens. We used the centroid size (CS) of the semilandmarks digitized on each semicircular canal as a proxy for canal size. Because the shape of the SCC often departs widely from circularity, as in Perier *et al*.^47^, we chose to use a metric designed to capture the size of complex geometry rather than the more commonly used semicircular canal radius of curvature. In a similar way, we used the centroid size (CS) of the semilandmarks digitized on the cochlea as a proxy for cochlear size. We also measured the angle between the three pairs of canals (and angular variance within the four groups), in order to quantify potential departure from orthogonality. Following Perier *et al*.^47^, shape variance (SV) was computed using the metrics proposed by Zelditch *et al*.^48^, which is identical to the trace of the variance-covariance matrix of the Procrustes data.

Permutation tests (10000 rounds) were used to compare the levels of variance (shape variance, CS variance, angular variance between pairs of semicircular canals) between dog groups. In order to compare values of SV between groups of dogs, as group specific mean shapes might differ, prior to performing the resampling procedure, for each specimen of a given dog group, we subtracted from its Procrustes coordinates the group-consensus to which it belongs. In a similar way, to allow for the comparison of CS Variance and CCL variance, we normalized CS and CCL for each dog group (to reach mean (CS) = 1 and mean (CCL) = 1) for all four dog groups before doing the resampling procedure. Statistical procedures were performed with R 3.3.3^49^.

Cochlear turns were counted after West^50^, where the inflection point at the round window is used as the starting point to count the number of half-turns up to the apex. For this purpose the inner ear models were oriented in a way so the cochlea was seen from above and a line projecting from the inflection point through the central axis of the turns was drawn to help estimation of the number of half-turns by counting the number of times the spiral path intersects this line^50^.

A recent and comprehensive study by Parker *et al*.^51^, has shown that the history of dogs is a network of hybridized breeds. It is thus not possible to apply within this single species phylogenetic correction as it is otherwise done in comparative analyses. Please also notice that most of the breeds are recent creations, postdating around 1850. Furthermore, the phylogenetic position of the numerous prehistoric dog samples examined in the network of dogs is unknown.

### Data availability

The 3D surface data are deposited in https://doi.org/10.18563/m3.3.4.e1.

## Results

**Figure 2:**
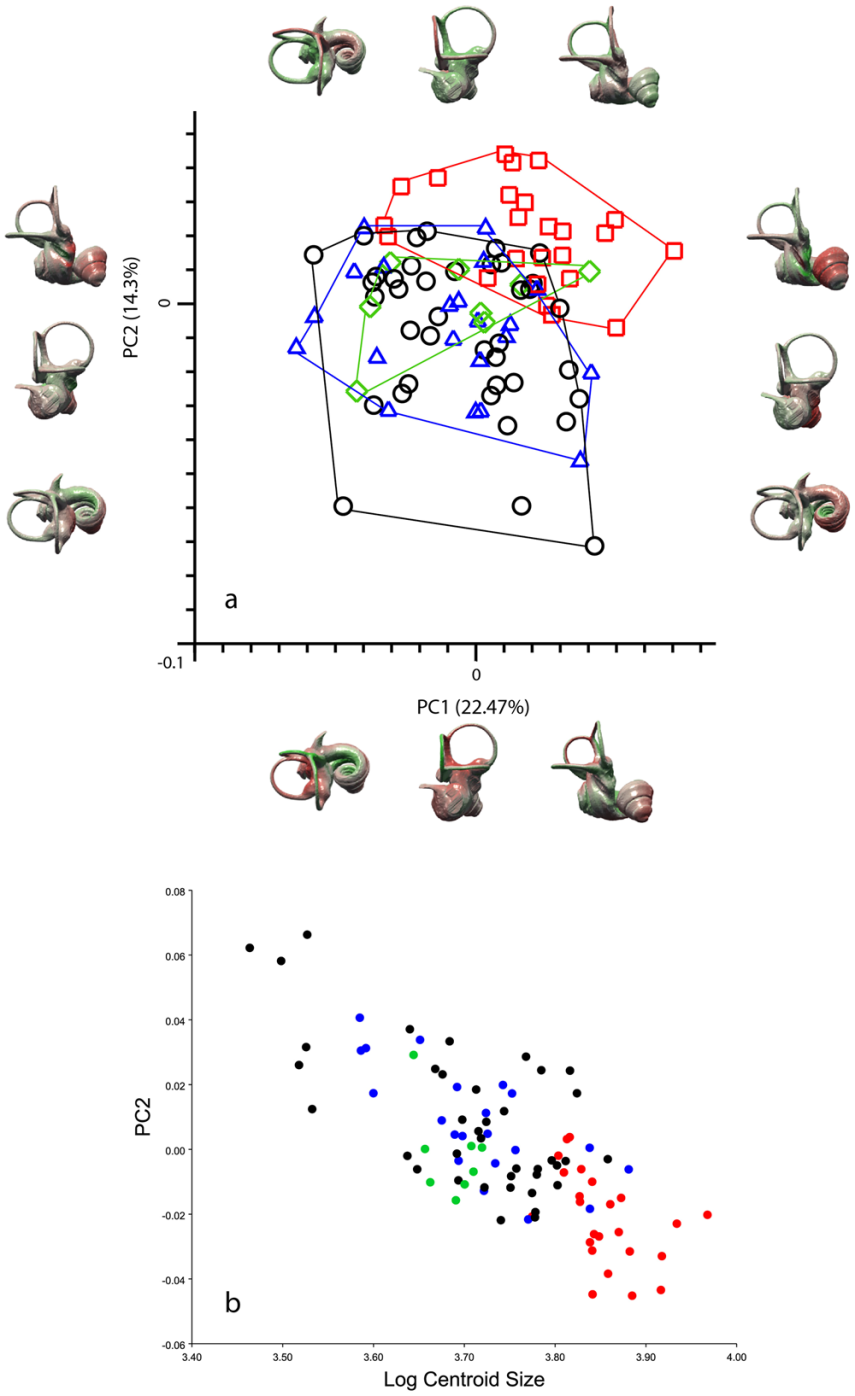
Principal component analysis bony labyrinth and shape regression. (**a**) Principal component analysis (PCA) performed on a set of 93 semilandmarks. Plot of PC1 (22.47%) and PC2 (14.3%) (red squares: wolves; blue triangles: ‘prehistoric’ specimens; black circles: dogs; green rhombi: dingoes) including morphological patterns associated with shape differentiation of the bony labyrinth on the first two axes (37%) in anterolateral, posterolateral and dorsal view. Deformations shown using the three-dimensional virtual endocast of the bony labyrinth of a wolf (ZMUZH_MAMM_20201) (green: negative displacement; red: positive displacement). From left to right, the three outliers at the bottom of the graph are a Chihuahua (TMM M-150), a pug (ZMUZH 10175) and a poodle (NMB 12079). (**b**) Shape regression of raw procrustes coordinates of principal component 2 against Log Centroid Size (red: wolves; blue: prehistoric dogs; black: modern dogs; green: dingoes).

Dogs occupy a larger and more differentiated morphospace than wolves, with little overlap between the two (Fig. 2a and Supplementary Figure 1). However, this result is probably the effect of only a few modern specimens which fall as outliers, as the difference in shape variance levels between modern breeds and wolves is not statistically significant (Table 1). Except for a minor overlap, the prehistoric dogs are also separated from wolves (Fig. 2a). The first three principal components account for 45.4% of the total variation. PC1 explains 22.5% of the variance, PC2 14.3% and PC3 explains 10.6% (Supplementary Fig. 2). Along PC1 the groups are distributed more or less across all values; the wolves have a tendency to slightly more positive values. The wolves are located at positive values for PC2 whereas the modern and prehistoric dogs are found at more negative values for PC2 (Fig. 2a). There is considerable overlap of the groups along PC3, and no clear separation along this axis (Supplementary Figure 3).

**Table 1:** Shape variance levels of the bony labyrinth.

	Wolf	Prehistoric_dog	Dingo	Dog
Whole_inner_ear	0.00184	0.00187	0.00158	0.00226
Cochlea	0.00760	0.00944	0.01243	0.00894
Canals	0.00328	0.00372	0.00254	0.00364
Lateral	0.00140	0.00151	0.00142	0.00204
Anterior	0.00205	0.00221	0.00140	0.00238
Posterior	0.00186	0.00211	0.00184	0.00287
		wolf	prehistoric dog	dingo
***Whole labyrinth***				
	prehistoric dog	0.93		
	dingo	0.43	0.51	
	modern dog	0.16	0.23	0.15
***Cochlea***				
	prehistoric dog	0.33		
	dingo	0.12	0.42	
	modern dog	0.3	0.75	0.17
***3 semicircular canals***				
	prehistoric dog	0.54		
	dingo	0.31	0.24	
	modern dog	0.31	0.93	0.2
***Lateral semicircular canal***				
	prehistoric dog	0.64		
	dingo	0.95	0.82	
	modern dog	0.065	0.17	0.32
***Anterior semicircular canal***				
	prehistoric dog	0.65		
	dingo	0.14	0.11	
	modern dog	0.3	0.61	0.054
***Posterior semicircular canal***				
	prehistoric dog	0.52		
	dingo	0.97	0.59	
	modern dog	0.036	0.12	0.19

Absolute levels of shape variance for the whole inner ear as well as the separate structures for the four groups (top) and differences of shape variance (SV) levels between the four groups for the whole bony labyrinth, only the cochlea, only the semicircular canals and the lateral, the anterior and the posterior semicircular canal separately (red = significant value).

Generally, morphological tranformations associated with the principal components are subtle. Along PC1, the shape of the semicircular canals does not change much, apart from a slight increase in size of the anterior semicircular canal and undulation in its superior aspect towards positive values, as well as a minor shape change of the lateral semicircular canal, from a more or less ovoid to a rounder shape towards positive values. Changes in the cochlea are more significant; while it is rather narrow and pointed at negative values for PC1, it gets broader towards positive values. This broadening is more prominent in the distal turns. There is only a weak allometric effect on PC1 (0.66%) whereas the allometric effect on PC2 is strong (57.71%) (Fig. 2b).

Along PC2, the anterior semicircular canal increases in size towards positive values, and it undulates in its superior portion. The shape of the posterior semicircular canal changes from dorsoventrally flattened at negative values to larger and less flattened at positive values. Also, the posterior semicircular canal is more wavy and the common crus elongates and inclines anteriorly towards positive values. The angle between the anterior and the lateral semicircular canal gets larger and the lateral canal increases in radius while its shape stays the same towards positive values for PC2. The lateral and the posterior semicircular canal are perpendicular to each other at negative values for PC2, whereas the angle between them increases slightly towards positive values. The cochlea on the other hand is broader and less pointed in shape at negative values.

With respect to the PCA performed on the subset of semilandmarks describing the three semicircular canals, prehistoric dogs are separated from wolves along PC1, whereas modern dogs exhibit more overlap with the wolves. The dingoes are clustered in the middle of the morphospace for all PC1-PC2 for the semicircular canals only (Supplementary Fig. 4).

Variation is by far the highest in the lateral semicircular canal, as revealed by PCA on individual canals. Shape variance (whole inner ear SV, cochlear SV, canals SV) levels are greater in modern dog breeds than in wolves (see Table 1 shape variance levels, the differences amount to between 11% greater up to 54% greater depending on the structure), but this difference is not statistically significant. The only significant difference in SV was found between the posterior canal of wolves and that of modern dogs (Table 1). Likewise, we found no significant difference between dog groups in the levels of angular variance between semicircular canal pairs. Only one test showed a significant difference: wolves express a significantly higher level of angular variance between the lateral and posterior semicircular canal angles than dogs (Supplementary Table 6).

Variance in centroid size levels of variance are in most cases significantly lower in wolves than in dogs and prehistoric dogs. Common crus length levels of variance are significantly lower in dingoes than in wolves and prehistoric dogs (Supplementary Table 7).

The number of cochlear turns across the whole sample varies from 3 to 3.5 (Supplementary Table 1). Except two specimens with 3.25, wolves exhibit 3.5 cochlear turns. Within the modern and the prehistoric dogs, all three values occur; 3 turns, however is the least common. In dingoes, 3 turns is slightly predominant over 3.25 turns.

## Discussion

Dogs exhibit an expanded occupation of inner ear morphospace than wolves, and these differences are largely size-related. In addition to the changes coupled with the initial phases of domestication, intense selective breeding has led to even more morphological disparity in dogs^52^, in particular in skull shape (e.g., Drake and Klingenberg^2^). This extent of morphological disparity does not extend to intricate details of the vestibular system. There is no statistical significant difference in the levels of shape variation between prehistoric and modern dogs. The shape variance in this sample is slightly higher for the different components of the inner ear in modern dogs than in wolves, but these differences are not significant. Contrary to our expectations, wolves express a significantly greater level of variance in the angle between the lateral and the posterior canal than domestic dog breeds. As a general trend, among the four groups, wolves express the highest levels of angular variance for all pairs of semicircular canals; but the differences, apart from the above-mentioned, are not statistically significant (Supplementary Table 6). As expected, wolves have smaller levels of size variation than dogs (Supplementary Table 7). That the variation in the lateral semicircular canal is the largest when examining the PCA on individual canals is not surprising: this is the variable zone in sloths^19^ and it is the insertion of the LSC that yields most phylogenetic information in ruminants^53^.

In terms of the shape of the semicircular canals, dingoes reflect the mean shape in the context of variation in the sample (Supplementary Fig. 4). This mirrors the condition of wild forms in other organ systems, in which there is an incomplete return to the characteristics of the ancestral form^16^.

### Markers of domestication

The difference between dog breeds and wolves are well-recorded, but distinguishing prehistoric dogs from wolves is a more difficult task, as morphological markers are restricted to relatively subtle changes in size and some aspects of the dentition^3,54^. Attribution of prehistoric remains to the domestic dog are challenging^55–57^. Although inner ear shape discriminates geographically distinct subspecies of some mammals^12^, it does not seem to provide an independent marker for domestication, a process that started at least some 14’000 years ago if not perhaps much earlier^55,58–60^, albeit significant morphological variability in dog size is only recorded around 15’000 years^61^.

### Disparity in inner ear structures

The morphology of highly conserved organs, such as the vestibular organ or the cochlea, may vary significantly when selection pressures are minimized due to a decrease in functional demand^19^. This general principle had already been noted by Darwin^62^, who stated that ‘an organ, when rendered useless, may well be variable, for its variations cannot be checked by natural selection’.

While the inner ear has been used to investigate locomotory capacities in a wide range of animals^10,13,26,63^, little information relates its shape to locomotory behaviours in closely related species with relatively homogeneous adaptations, phylogeny playing here often a predominant role^20,53^. Grohé^21^ reported on subtle shape differences among carnivorans, particularly in the anterior canal in semi-aquatic musteloids versus terrestrial forms of the same clade for two very different locomotory abilities (swimming and cursorial).

In spite of the variation in the gait parameters of dogs, as documented in a comprehensive study involving 32 different dog breeds^64^, the basic locomotory pattern is similar, and is thus unlikely to be detected in semi-circular canal shape as observed here. The requirements for coordinating fast and complex movements in a 3D space (i.e. the function of the semicircular canals) are similar among all dogs. The differences in locomotion that have been hypothesized to result in variation in semicircular canal morphology are of a much larger kind than those characteristic of dogs^65^. The conservatism in shape of the inner ear contrasts with the variation in the number of cochlear turns, which is higher in dogs and dingoes than in wolves. Fleischer^15^ reported on different average values in several parameters of middle ear and cochlear proportions between wolves and domesticated dogs belonging to three breeds, each group consisting of six individuals. This anatomical area deserves further examination.

The bony labyrinth changes during domestication recorded here are largely size-related. The isolation of dingoes for thousands of years prior to colonization^66,67^ did not lead to subsequent changes in morphology towards the ‘undomesticated’ state. An analogous trend was found in studies of brain size. Brain size was reduced by 28.8% upon domestication in dogs compared to wolves, in addition to being more variable, whereas encephalization of dingoes, remained at the level of dogs^18^.

## Conclusion

The inclusion of individuals from different time periods and geographic areas in our extensive study and documentation of a complex structure, support the generalisations made in this work. These concern the characterization of the inner ear in wolves, dogs and dingoes. ‘Dogs’ included both prehistoric forms and well as a sample of different breeds that exemplify the spectrum of variation in these animals. Advances of genomic studies of dog domestication have benefited from population sampling and data complexity^14^ – a similar trend in phenomic studies^65^ can provide new insights into the domestication process.

Contrary to expectations based on the great disparity documented in skull shape of domesticated dogs, their inner ear is much conserved in shape and the larger morphospace occupation in comparison with wolves is correlated mostly with differences in size. Morphological disparity is generated by scaling, consistent with other studies that have promoted the role of size as a line of evolutionary least resistance^68^.

## Electronic supplementary material

Supplementary 7 tables and 4 figures
