# Supplementary material for: Size Variation under Domestication: Conservatism in the inner ear shape of wolves, dogs and dingoes
Source: Sci Rep. 2017 Oct 17;7:13330. doi: 10.1038/s41598-017-13523-9 (PMC5645459; doi:10.1038/s41598-017-13523-9)

# Supplementary Information

**Size Variation under Domestication: Conservatism in the inner ear shape of wolves, dogs and dingoes**

Anita V. Schweizer, Paläontologisches Institut und Museum, Karl-Schmid-Strasse 4, 8006 Zürich, Switzerland. anita.schweizer2@gmail.com

Renaud Lebrun, Laboratoire de Paléontologie, Institut des Sciences de l'Evolution de Montpellier, UMR-CNRS 5554, cc 064, Université de Montpellier 2, 34095 Montpellier Cedex 5, France. renaud.lebrun@umontpellier.fr

Laura A. B. Wilson, PANGEA Research Centre, School of Biological, Earth and Environmental Sciences, University of New South Wales, Sydney, NSW 2052, Australia. Email: laura.wilson@unsw.edu.au

Loïc Costeur, Naturhistorisches Museum Basel, Augustinergasse 2, 4051 Basel, Switzerland. loic.costeur@bs.ch

Thomas Schmelzle, Fleckensteinstraße 7, 74206 Bad Wimpfen. dr.thomas.schmelzle@gmx.de

* Marcelo R. Sánchez-Villagra, Paläontologisches Institut und Museum, Karl-Schmid-Strasse 4, 8006 Zürich, Switzerland. [m.sanchez@pim.uzh.ch](mailto:m.sanchez@pim.uzh.ch)

**Table 1 List of the examined specimens and number of cochlear turns**

List of specimens including breed, numbers of turns of the cochlea, age and cranial base length (red: wolves; blue: prehistoric dogs; black: modern dogs; green: dingoes).

|  | ID | genus | species | name | cochlear turns | age | cranial base [mm] |
| --- | --- | --- | --- | --- | --- | --- | --- |
| 1 | ZMUZH 17603 | *Canis* | *lupus chanco* | Tibetan wolf | 3.5 | 2 years | 62 |
| 2 | ZMUZH 20201 | *Canis* | *lupus chanco* | Tibetan wolf | 3.5 | 13 days | N/A |
| 3 | ZMUZH 17602 | *Canis* | *lupus chanco* | Tibetan wolf | 3.5 | 2 years | 62 |
| 4 | ZMUZH 13854 | *Canis* | *lupus* | Grey Wolf | 3.5 | adult | 75 |
| 5 | NMB 8381 | *Canis* | *lupus* | Wolf | 3.5 | subadult | 58 |
| 6 | NMB SCM320 | *Canis* | *lupus* | Wolf | 3.5 | adult | 70 |
| 7 | NMB Z309 | *Canis* | *lupus* | Wolf | 3.25 | adult | 65 |
| 8 | NMB 2761 | *Canis* | *lupus* | Wolf | 3.5 | adult | 74 |
| 9 | M39960 | *Canis* | *lupus* | Wolf | 3.5 |  |  |
| 10 | NMB 5258 | *Canis* | *lupus* | Wolf | 3.5 | juvenile | N/A |
| 11 | NMB 8635 | *Canis* | *lupus* | Wolf | 3.5 | adult | N/A |
| 12 | ZMUZH 20202 | *Canis* | *lupus chanco* | Tibetan wolf | 3.5 | 13 days | N/A |
| 13 | NMB C. 1362 | *Canis* | *lupus lycaon* | Timberwolf | 3.5 | adult | 69 |
| 14 | NMB No Nb | *Canis* | *lupus occidentalis* | Mackenzie Wolf | 3.5 | adult | 81 |
| 15 | NMB 11019 | *Canis* | *lupus arabs* | Arabischer Wolf | 3.25 | adult | 62 |
| 16 | ZMUZH 17118 | *Canis* | *lupus* | Wolf | 3.5 | 8 months | 68 |
| 17 | ZMUZH 15858 | *Canis* | *lupus* | Wolf | 3.5 | 10 years | 68 |
| 18 | ZMUZH 17612 | *Canis* | *lupus chanco* | Tibetan wolf | 3.5 | 6 years | 63 |
| 19 | ZMUZH 18082 | *Canis* | *lupus chanco* | Tibetan wolf | 3.5 | 10 years | 67 |
| 20 | ZMUZH 17210 | *Canis* | *lupus occidentalis* | Mackenzie Wolf | 3.5 | 16 years | 72 |
| 21 | UMZC K.3141 | *Canis* | *lupus* | Wolf | 3.5 |  |  |
| 22 | UMZC K.3150.1 | *Canis* | *lupus* | Wolf | 3.5 | juvenile | |
| 23 | UMZC K.3152 | *Canis* | *lupus* | Wolf | 3.5 | adult | |
| 24 | UMZC K.3149 | *Canis* | *lupus* | Wolf | 3.5 |  |  |
| 25 | NMB A.403. | *Canis* | *lupus familiaris* | prehistoric dog | 3.25 |  | 47 |
| 26 | NMB A.5.a. | *Canis* | *lupus familiaris* | prehistoric dog | 3.25 |  | 48 |
| 27 | ZMUZH A.II. | *Canis* | *lupus familiaris* | prehistoric dog | 3.25 |  |  |
| 28 | ZMUZH A.VIII. | *Canis* | *lupus familiaris* | prehistoric dog | 3.5 |  |  |
| 29 | ZMUZH We.6. | *Canis* | *lupus familiaris* | prehistoric dog | 3.5 |  | 58 |
| 30 | ZMUZH Ez.2. | *Canis* | *lupus familiaris* | prehistoric dog | 3.25 |  | N/A |
| 31 | ZMUZH Ez.E. | *Canis* | *lupus familiaris* | prehistoric dog | 3.5 |  | N/A |
| 32 | ZMUZH A.6. | *Canis* | *lupus familiaris* | prehistoric dog | 3.5 |  |  |
| 33 | ZMUZH Wyn.9. | *Canis* | *lupus familiaris* | prehistoric dog | 3.5 |  | 67 |
| 34 | ZMUZH F.48. | *Canis* | *lupus familiaris* | prehistoric dog | 3.5 |  | 58 |
| 35 | ZMUZH Terp.1. | *Canis* | *lupus familiaris* | prehistoric dog | 3.25 |  | 59 |
| 36 | ZMUZH A.VII. | *Canis* | *lupus familiaris* | prehistoric dog | 3.5 |  |  |
| 37 | ZMUZH A.VI. | *Canis* | *lupus familiaris* | prehistoric dog | 3.5 |  |  |
| 38 | ZMUZH A.IV. | *Canis* | *lupus familiaris* | prehistoric dog | 3 |  |  |
| 39 | NMBE-DUR-1124 | *Canis* | *lupus familiaris* | prehistoric dog | 3.5 |  | 70 |
| 40 | NMBE-LUS-1095 | *Canis* | *lupus familiaris* | prehistoric dog | 3.5 |  | 50 |
| 41 | NMBE-LUS-1102 | *Canis* | *lupus familiaris* | prehistoric dog | 3.5 |  | 44 |
| 42 | NMBE-BUR-1057 | *Canis* | *lupus familiaris* | prehistoric dog | 3.5 |  | 40 |
| 43 | NMBE-SUT-1119 | *Canis* | *lupus familiaris* | prehistoric dog | 3.25 |  | 42 |
| 44 | NMBE 16 | *Canis* | *lupus familiaris* | prehistoric dog | 3.25 |  | N/A |
| 45 | NMBE-LAT-1136 | *Canis* | *lupus familiaris* | prehistoric dog | 3.25 |  | 57 |
| 46 | SZ 7961 | *Canis* | *lupus familiaris* | Chow chow | 3.5 | 15 years | |
| 47 | SZ 7959 | *Canis* | *lupus familiaris* | Chow chow | 3.5 | 11 years | |
| 48 | SZ 7958 | *Canis* | *lupus familiaris* | Chow chow | 3.25 | 7 years | |
| 49 | NMBE 1051209 | *Canis* | *lupus familiaris* | Afghan Greyhound | 3.5 | adult | 71 |
| 50 | NMBE 1051226 | *Canis* | *lupus familiaris* | Afghan Greyhound | 3.5 | juvenile | N/A |
| 51 | NMBE 1051381 | *Canis* | *lupus familiaris* | Akita Inu | 3.5 | adult | 63 |
| 52 | NMBE 1051418 | *Canis* | *lupus familiaris* | Akita Inu | 3.25 | juvenile | N/A |
| 53 | PIMUZ A/V 2835 | *Canis* | *lupus familiaris* | German Shepherd | 3.25 |  | 60 |
| 54 | PIMUZ A/V 2837 | *Canis* | *lupus familiaris* | German Shepherd | 3.25 | 11 years | 64 |
| 55 | PIMUZ A/V 2834 | *Canis* | *lupus familiaris* | Appenzeller Mountain Dog | 3.25 | 11.5 years | 60 |
| 56 | PIMUZ A/V 2845 | *Canis* | *lupus familiaris* | Dachshund | 3.25 | 1.5 years | 44 |
| 57 | PIMUZ A/V 2831 | *Canis* | *lupus familiaris* | Dachshund | 3 | 15 years | 50 |
| 58 | ZMUZH 17712 | *Canis* | *lupus familiaris* | Northafrican Greyhound (Sloughi) | 3.25 |  | 58 |
| 59 | ZMUZH 17713 | *Canis* | *lupus familiaris* | Russian greyhound (Borzoi) | 3.25 | juvenile | 45 |
| 60 | SZ 7930 | *Canis* | *lupus familiaris* | Cocker Spaniel | 3.25 | 13 years | |
| 61 | SZ 7929 | *Canis* | *lupus familiaris* | Cocker Spaniel | 3.25 | 2.5 years | |
| 62 | SZ 7926 | *Canis* | *lupus familiaris* | Cocker Spaniel | 3.5 | 11 years | |
| 63 | NMB 12080 | *Canis* | *lupus familiaris* | Labrador Retriever | 3 |  | 59 |
| 64 | NMB 12081 | *Canis* | *lupus familiaris* | Labrador Retriever | 3.5 |  | 65 |
| 65 | NMB 12079 |  | *lupus familiaris* | Poodle | 3.5 |  | 42 |
| 66 | NMB 12078 | *Canis* | *lupus familiaris* | Poodle | 3.25 |  | 39 |
| 67 | PIMUZ A/V 3001 | *Canis* | *lupus familiaris* | Foxterrier | 3.25 | 13 years | 50 |
| 68 | PIMUZ A/V 2832 | *Canis* | *lupus familiaris* | Foxterrier | 3.25 | 14.5 years | 45 |
| 69 | ZMUZH 10166 | *Canis* | *lupus familiaris* | Pug | 3.5 |  | 37 |
| 70 | ZMUZH 10175 | *Canis* | *lupus familiaris* | Pug | 3.5 |  | 36 |
| 71 | PIMUZ A/V 3000 | *Canis* | *lupus familiaris* | Boxer | 3.25 |  | 55 |
| 72 | PIMUZ A/V 2847 | *Canis* | *lupus familiaris* | Boxer | 3.5 |  | 56 |
| 73 | PIMUZ A/V 2846 | *Canis* | *lupus familiaris* | Boxer | 3.5 |  | 58 |
| 74 | PIMUZ A/V 2836 | *Canis* | *lupus familiaris* | Boxer | 3.25 |  | 53 |
| 75 | ZMUZH 14842 | *Canis* | *lupus familiaris* | St. Bernard | 3.5 |  | 68 |
| 76 | ZMUZH 10342 | *Canis* | *lupus familiaris* | St. Bernard | 3.25 | adult | 71 |
| 77 | ZMUZH 10343 | *Canis* | *lupus familiaris* | St. Bernard | 3.25 | adult | 68 |
| 78 | TMM M-150 | *Canis* | *lupus familiaris* | Chihuahua | 3.25 |  |  |
| 79 | ZMUZH 13766 | *Canis* | *lupus familiaris* | Eskimo Dog | 3.5 |  | 63 |
| 80 | ZMUZH 17717 | *Canis* | *lupus familiaris* | Maremma Sheepdog | 3.5 |  | 64 |
| 81 | ZMUZH 17711 | *Canis* | *lupus familiaris* | Ibizan Hound | 3.25 | 7 months | 64 |
| 82 | ZMUZH 17714 | *Canis* | *lupus familiaris* | Caucasian Shepherd Dog | 3.25 |  | 60 |
| 83 | ZMUZH 17715 | *Canis* | *lupus familiaris* | Caucasian Shepherd Dog | 3.5 |  | 60 |
| 84 | UMZC K.3016 | *Canis* | *lupus familiaris* | | 3.5 |  |  |
| 85 | UNSW Z354 | *Canis* | *lupus dingo* | Dingo | 3.25 |  | 75.27 |
| 86 | UniSyd FVS #45 | *Canis* | *lupus dingo* | Dingo | 3 |  |  |
| 87 | M6297 | *Canis* | *lupus dingo* | Dingo | 3.25 |  |  |
| 88 | M24153 | *Canis* | *lupus dingo* | Dingo | 3 |  |  |
| 89 | M33608 | *Canis* | *lupus dingo* | Dingo | 3 |  |  |
| 90 | M38587 | *Canis* | *lupus dingo* | Dingo | 3 |  |  |
| 91 | UMZC K.3221 | *Canis* | *lupus dingo Blumenbach* | Dingo | 3 |  |  |
| 92 | UMZC K.3223 | *Canis* | *lupus dingo Blumenbach* | Dingo | 3.25 |  |  |

**Table 2 Geological age and finding site of prehistoric specimens**

Geological age of the prehistoric specimens as estimated based either on the finding site or 14-C dating.

| ID | geological age | finding site |
| --- | --- | --- |
| NMB A.403. | 5000-3000 BC | Insel Wird, Bodensee |
| NMB A.5.a. | 3000 BC | Wangen, Bodensee |
| ZMUZH A.II. | 1300-800 BC | Zürich, Alpenquai |
| ZMUZH A.VII. | 1300-800 BC | Zürich, Alpenquai |
| ZMUZH We.6. |  | NL |
| ZMUZH Ez.2. | 4300-2500 BC | Egolzwil, LU |
| ZMUZH Ez.E. | 4300-2500 BC | Egolzwil, LU |
| ZMUZH A.6. | 1300-800 BC | Zürich, Alpenquai |
| ZMUZH Wyn.9. |  | Terpen NL |
| ZMUZH F.48. |  | NL |
| ZMUZH Terp.1. |  | Terpen Groningen NL |
| ZMUZH A.VII. | 1300-800 BC | Zürich, Alpenquai |
| ZMUZH A.VI. | 1300-800 BC | Zürich, Alpenquai |
| ZMUZH A.IV. | 1300-800 BC | Zürich, Alpenquai |
| NMBE-DUR-1124 | 7542-7256 BC (14-C dated) | Dürriloch BE |
| NMBE-LUS-1095 | 4000-500 BC | Lüscherz BE |
| NMBE-LUS-1102 | 4000-500 BC | Lüscherz BE |
| NMBE-BUR-1057 | 4000-3500 BC | Burgäschisee-Süd BE |
| NMBE-SUT-1119 | 4000-500 BC | Sutz-Lattrigen BE |
| NMBE 16 | 4000-500 BC | Robenhausen ZH |
| NMBE-LAT-1136 | 250-50 BC | LaTene NE |

**Table 3 CT scanning facilities**

Institutions and facilities in which skulls were scanned using high-resolution x-ray micro-computed tomography (CT) and respective scanner type.

| Institution | Scanner |
| --- | --- |
| Giesserei Technologie Aalen (GTA), Fachhochschule Aalen, Germany | RayScan200 and v\|tome\|x s |
| SCANCO Medical AG, Brüttisellen, Switzerland | XtremeCT II |
| Biomaterials Science Center (BMC), University of Basel, Switzerland | phoenix nanotom m |
| Biological Resources Imaging Laboratory (BRIL), Mark Wainwright Analytical Centre, University of New South Wales (UNSW), Australia | Siemens Inveon MicroPET-CT |
| Cambridge Biotomography Centre, England | Nikon XT H 225 ST |
| Paleontological Institute and Museum, University of Zurich, Switzerland | Nikon XT H 225 ST |

**Table 4 Scan parameters**

Parameters used for the CT Scanning of the individual specimens, including resolution in µm, scanner type, voltage in kV, current in µA, and type of filter.

|  | ID | scanner | resolution [mm] | kV | uA | filter |
| --- | --- | --- | --- | --- | --- | --- |
| 1 | ZMUZH 17603 | v\|tome\|x s | 0.015 |  |  |  |
| 2 | ZMUZH 20201 | RayScan200 | 0.05 |  |  |  |
| 3 | ZMUZH 17602 | XtremeCT II | 0.08 |  |  |  |
| 4 | ZMUZH 13854 | RayScan200 | 0.07 |  |  |  |
| 5 | NMB 8381 | XtremeCT II | 0.08 |  |  |  |
| 6 | NMB SCM320 | XtremeCT II | 0.08 |  |  |  |
| 7 | NMB Z309 | XtremeCT II | 0.08 |  |  |  |
| 8 | NMB 2761 | XtremeCT II | 0.08 |  |  |  |
| 9 | M39960 | Siemens Inveon MicroPET-CT | 0.071 |  |  |  |
| 10 | NMB 5258 | XtremeCT II | 0.08 |  |  |  |
| 11 | NMB 8635 | XtremeCT II | 0.08 |  |  |  |
| 12 | ZMUZH 20202 | XT H 255ST | 0.046 |  |  |  |
| 13 | NMB C. 1362 | XtremeCT II | 0.08 |  |  |  |
| 14 | NMB No Nb | XtremeCT II | 0.08 |  |  |  |
| 15 | NMB 11019 | XtremeCT II | 0.08 |  |  |  |
| 16 | ZMUZH 17118 | XT H 255 ST | 0.05 | 212 | 201 |  |
| 17 | ZMUZH 15858 | XT H 255 ST | 0.05 | 213 | 201 |  |
| 18 | ZMUZH 17612 | XT H 255 ST | 0.05 |  |  |  |
| 19 | ZMUZH 18082 | XT H 255 ST | 0.055 | 165 | 115 | 0.25mm Cu |
| 20 | ZMUZH 17210 | XT H 255 ST | 0.06 | 217 | 190 |  |
| 21 | UMZC K.3141 | XT H 255 ST | 0.029 |  |  |  |
| 22 | UMZC K.3150.1 | XT H 255 ST | 0.033 |  |  |  |
| 23 | UMZC K.3152 | XT H 255 ST | 0.027 |  |  |  |
| 24 | UMZC K.3149 | XT H 255 ST | 0.03 |  |  |  |
| 25 | NMB A.403. | XtremeCT II | 0.067 |  |  |  |
| 26 | NMB A.5.a. | XtremeCT II | 0.067 |  |  |  |
| 27 | ZMUZH A.II. | XT H 255 ST | 0.05 | 164 | 186 |  |
| 28 | ZMUZH A.VIII. | XT H 255 ST | 0.05 | 165 | 230 | 0.1mm Al |
| 29 | ZMUZH We.6. | XT H 255 ST | 0.05 | 200 | 205 |  |
| 30 | ZMUZH Ez.2. | XT H 255 ST | 0.05 | 175 | 200 |  |
| 31 | ZMUZH Ez.E. | XT H 255 ST | 0.05 | 125 | 170 |  |
| 32 | ZMUZH A.6. | XT H 255 ST | 0.057 | 160 | 230 |  |
| 33 | ZMUZH Wyn.9. | XT H 255 ST | 0.055 | 220 | 235 |  |
| 34 | ZMUZH F.48. | XT H 255 ST | 0.05 | 220 | 235 |  |
| 35 | ZMUZH Terp.1. | XT H 255 ST | 0.05 | 170 | 250 |  |
| 36 | ZMUZH A.VII. | XT H 255 ST | 0.05 | 190 | 100 |  |
| 37 | ZMUZH A.VI. | XT H 255 ST | 0.052 | 195 | 210 | 0.1mm Cu |
| 38 | ZMUZH A.IV. | XT H 255 ST | 0.055 | 165 | 230 |  |
| 39 | NMBE-DUR-1124 | XT H 255 ST | 0.05 | 223 | 190 | 0.1mm Cu |
| 40 | NMBE-LUS-1095 | XT H 255 ST | 0.05 | 224 | 170 | 0.1mm Cu |
| 41 | NMBE-LUS-1102 | XT H 255 ST | 0.05 | 190 | 160 | 0.1mm Cu |
| 42 | NMBE-BUR-1057 | XT H 255 ST | 0.05 | 182 | 160 | 0.1mm Cu |
| 43 | NMBE-SUT-1119 | XT H 255 ST | 0.05 | 215 | 190 | 0.1mm Cu |
| 44 | NMBE 16 | XT H 255 ST | 0.05 | 190 | 180 | 0.1 mm Cu |
| 45 | NMBE-LAT-1136 | XT H 255 ST | 0.05 |  |  |  |
| 46 | SZ 7961 | v\|tome\|x s | 0.012 |  |  |  |
| 47 | SZ 7959 | v\|tome\|x s | 0.015 |  |  |  |
| 48 | SZ 7958 | v\|tome\|x s | 0.012 |  |  |  |
| 49 | NMBE 1051209 | RayScan200 | 0.05 |  |  |  |
| 50 | NMBE 1051226 | RayScan200 | 0.06 |  |  |  |
| 51 | NMBE 1051381 | RayScan200 | 0.06 |  |  |  |
| 52 | NMBE 1051418 | RayScan200 | 0.05 |  |  |  |
| 53 | PIMUZ A/V 2835 | XtremeCT II | 0.091 | 68 | 1470 |  |
| 54 | PIMUZ A/V 2837 | XtremeCT II | 0.091 | 68 | 1470 |  |
| 55 | PIMUZ A/V 2834 | XtremeCT II | 0.091 | 68 | 1470 |  |
| 56 | PIMUZ A/V 2845 | XtremeCT II | 0.091 | 68 | 1470 |  |
| 57 | PIMUZ A/V 2831 | XtremeCT II | 0.091 | 68 | 1470 |  |
| 58 | ZMUZH 17712 | v\|tome\|x s | 0.011 |  |  |  |
| 59 | ZMUZH 17713 | v\|tome\|x s | 0.011 |  |  |  |
| 60 | SZ 7930 | v\|tome\|x s | 0.013 |  |  |  |
| 61 | SZ 7929 | v\|tome\|x s | 0.012 |  |  |  |
| 62 | SZ 7926 | v\|tome\|x s | 0.015 |  |  |  |
| 63 | NMB 12080 | phoenix nanotom m | 0.04 |  |  |  |
| 64 | NMB 12081 | XtremeCT II | 0.091 | 68 | 1470 |  |
| 65 | NMB 12079 | phoenix nanotom m | 0.03 |  |  |  |
| 66 | NMB 12078 | XtremeCT II | 0.091 | 68 | 1470 |  |
| 67 | PIMUZ A/V 3001 | XtremeCT II | 0.091 | 68 | 1470 |  |
| 68 | PIMUZ A/V 2832 | XtremeCT II | 0.091 | 68 | 1470 |  |
| 69 | ZMUZH 10166 | v\|tome\|x s | 0.011 |  |  |  |
| 70 | ZMUZH 10175 | v\|tome\|x s | 0.014 |  |  |  |
| 71 | PIMUZ A/V 3000 | v\|tome\|x s | 0.0153 |  |  |  |
| 72 | PIMUZ A/V 2847 | v\|tome\|x s | 0.0135 |  |  |  |
| 73 | PIMUZ A/V 2846 | v\|tome\|x s | 0.0225 |  |  |  |
| 74 | PIMUZ A/V 2836 | v\|tome\|x s | 0.0175 |  |  |  |
| 75 | ZMUZH 14842 | v\|tome\|x s | 0.0175 |  |  |  |
| 76 | ZMUZH 10342 | v\|tome\|x s | 0.0175 |  |  |  |
| 77 | ZMUZH 10343 | v\|tome\|x s | 0.02 |  |  |  |
| 78 | TMM M-150 |  | 0.14 |  |  |  |
| 79 | ZMUZH 13766 | XT H 255 ST | 0.046 |  |  |  |
| 80 | ZMUZH 17717 | XT H 255 ST | 0.05 |  |  |  |
| 81 | ZMUZH 17711 | XT H 255 ST | 0.05 | 146 | 165 |  |
| 82 | ZMUZH 17714 | XT H 255 ST | 0.056 |  |  |  |
| 83 | ZMUZH 17715 | XT H 255 ST | 0.05 |  |  |  |
| 84 | UMZC K.3016 | XT H 255 ST | 0.026 |  |  |  |
| 85 | UNSW Z354 | Siemens Inveon MicroPET-CT | 0.071 |  |  |  |
| 86 | UniSyd FVS #45 | Siemens Inveon MicroPET-CT | 0.071 |  |  |  |
| 87 | M6297 | Siemens Inveon MicroPET-CT | 0.071 |  |  |  |
| 88 | M24153 | Siemens Inveon MicroPET-CT | 0.071 |  |  |  |
| 89 | M33608 | Siemens Inveon MicroPET-CT | 0.071 |  |  |  |
| 90 | M38587 | Siemens Inveon MicroPET-CT | 0.071 |  |  |  |
| 91 | UMZC K.3221 | XT H 255 ST | 0.027 |  |  |  |
| 92 | UMZC K.3223 | XT H 255 ST | 0.031 |  |  |  |

**Table 5 Description of the starting and ending points for each of the five curves**

ASC: anterior semicircular canal; LSC: lateral semicircular canal; PSC: posterior semicircular canal; CCR: common crus. For the cochlea, the first three turns were digitized, since all of the specimens possess three or more cochlear turns (Perier et al. 2015).

| Curve | Starting point | Ending point |
| --- | --- | --- |
| 1 - cochlea | line through apex and inflection point at round window as in West (1985) | Completion of 3^rd^ turn on the line |
| 2 - LSC | Middle of the ampulla of the LSC | Point where the posterior end of the LSC meets the vestibule |
| 3 - ASC | Middle of the ampulla of the ASC | Y-junction between ASC, PSC & CCR |
| 4 - PSC | Middle of the ampulla of the PSC | Y-junction between ASC, PSC & CCR |
| 5 - CCR | Y-junction between ASC, PSC & CCR | Point where the ventral part of the CCR meets the vestibule |

**Table 6 Angular levels of variance**

Absolute values of mean angles and angular variance fot eh four groups and levels of angular variance of the angles between each pair of semicircular canals (ASC: anterior semicircualar canal LSC: lateral semicircualar canal; PSC: posterior semicircualar canal; red=significant values)

|  | Wolf | Prehistoric_dog | Dingo | Dog |
| --- | --- | --- | --- | --- |
| Mean(lat_post) ° | **93.02** | **89.45** | **88.63** | **89.42** |
| Var(lat_post) | 14.97 | 11.95 | 4.27 | 6.27 |
| Mean(Lat_ant) ° | **82.96** | **81.50** | **78.56** | **81.58** |
| Var(lat_ant) | 12.45 | 12.06 | 9.94 | 9.21 |
| Mean(ant_post) ° | **92.05** | **96.04** | **90.66** | **96.41** |
| Var(ant_post) | 19.25 | 17.99 | 13.63 | 17.28 |

|  |  | wolf | prehistoric dog | dingo |
| --- | --- | --- | --- | --- |
| *Angle between LSC & PSC* | |  |  |  |
|  | **prehistoric dog** | 0.73 |  |  |
|  | **dingo** | 0.35 | 0.26 |  |
|  | **modern dog** | 0.048 | 0.91 | 0.55 |
| *Angle between LSC & ASC* | |  |  |  |
|  | **prehistoric dog** | 0.95 |  |  |
|  | **dingo** | 0.76 | 0.74 |  |
|  | **modern dog** | 0.4 | 0.41 | 0.88 |
| *Angle between ASC & PSC* | |  |  |  |
|  | **prehistoric dog** | 0.85 |  |  |
|  | **dingo** | 0.53 | 0.65 |  |
|  | **modern dog** | 0.74 | 0.91 | 0.69 |

**Table 7 Centroid size levels of variance**

Absolute mean values of centroid size for the different structures and absolute values for centroid size variance (top) and levels of centroid size variance between the four groups on the whole bony labyrinth, only the cochlea, only the semicircular canals and the lateral, the anterior and the posterior semicircular canal separately (red=significant values).

|  | Wolf | Prehistoric_dog | Dingo | Dog |
| --- | --- | --- | --- | --- |
| Mean(centroid_size) | **47.25** | **41.02** | **40.24** | **40.89** |
| Var(Norm(centroid_size)) | 0.0021 | 0.0066 | 0.0013 | 0.0094 |
| Mean(centroid_size cochlea) | **9.40** | **8.52** | **8.39** | **8.41** |
| Var(Norm(centroid_size Cochlea)) | 0.0018 | 0.0045 | 0.0005 | 0.0064 |
| Mean(centroid_size canals) | **30.35** | **25.93** | **25.60** | **25.73** |
| Var(Norm(centroid_size canals)) | 0.0027 | 0.0087 | 0.0020 | 0.0130 |
| Mean(centroid_size lateral) | **12.38** | **10.77** | **10.52** | **10.49** |
| Var(Norm(centroid_size lateral)) | 0.0030 | 0.0083 | 0.0030 | 0.0146 |
| Mean(centroid_size anterior) | **11.67** | **9.87** | **9.87** | **9.84** |
| Var(Norm(centroid_size anterior)) | 0.0030 | 0.0096 | 0.0022 | 0.0160 |
| Mean(centroid_size posterior) | **10.88** | **8.98** | **8.86** | **8.93** |
| Var(Norm(centroid_size posterior)) | 0.0086 | 0.0156 | 0.0044 | 0.0133 |
| Mean(CCL in mm) | **3.64** | **3.07** | **2.93** | **3.03** |
| Var(Norm(CCL)) | 0.0130 | 0.0160 | 0.0015 | 0.0237 |

|  |  | wolf | prehistoric dog | dingo |
| --- | --- | --- | --- | --- |
| *Centroid size variance whole labyrinth* | |  |  |  |
|  | **prehistoric dog** | 0.026 |  |  |
|  | **dingo** | 0.54 | 0.15 |  |
|  | **modern dog** | 0.004 | 0.36 | 0.06 |
| *Centroid size variance cochlea* | |  |  |  |
|  | **prehistoric dog** | 0.64 |  |  |
|  | **dingo** | 0.22 | 0.08 |  |
|  | **modern dog** | 0.024 | 0.45 | 0.09 |
| *Centroid size variance 3 semicircular canals* | |  |  |  |
|  | **prehistoric dog** | 0.02 |  |  |
|  | **dingo** | 0.61 | 0.15 |  |
|  | **modern dog** | 0.004 | 0.33 | 0.08 |
| *Centroid size variance lateral semicircular canal* | | |  |  |
|  | **prehistoric dog** | 0.07 |  |  |
|  | **dingo** | 0.99 | 0.35 |  |
|  | **modern dog** | 0.0035 | 0.2 | 0.11 |
| *Centroid size variance anterior semicircular canal* | | |  |  |
|  | **prehistoric dog** | 0.03 |  |  |
|  | **dingo** | 0.66 | 0.16 |  |
|  | **modern dog** | 0.009 | 0.27 | 0.87 |
| *Centroid size variance posterior semicircular canal* | | |  |  |
|  | **prehistoric dog** | 0.16 |  |  |
|  | **dingo** | 0.33 | 0.17 |  |
|  | **modern dog** | 0.33 | 0.69 | 0.29 |
| *Length variance common crus* | |  |  |  |
|  | **prehistoric dog** | 0.54 |  |  |
|  | **dingo** | 0.04 | 0.04 |  |
|  | **modern dog** | 0.39 | 0.58 | 0.15 |

**Figure 1 Principal component analysis of 20 different modern dog breeds**

Principal component analysis on the whole bony labyrinth with numbers indicating the location of the different dog specimens and thus breeds in the morphospace. Numbers correspond to ID in Suppelmentary table 1 (squares: wolves; triangles: 'prehistoric' specimens; circles: dogs; rhombi: dingoes).


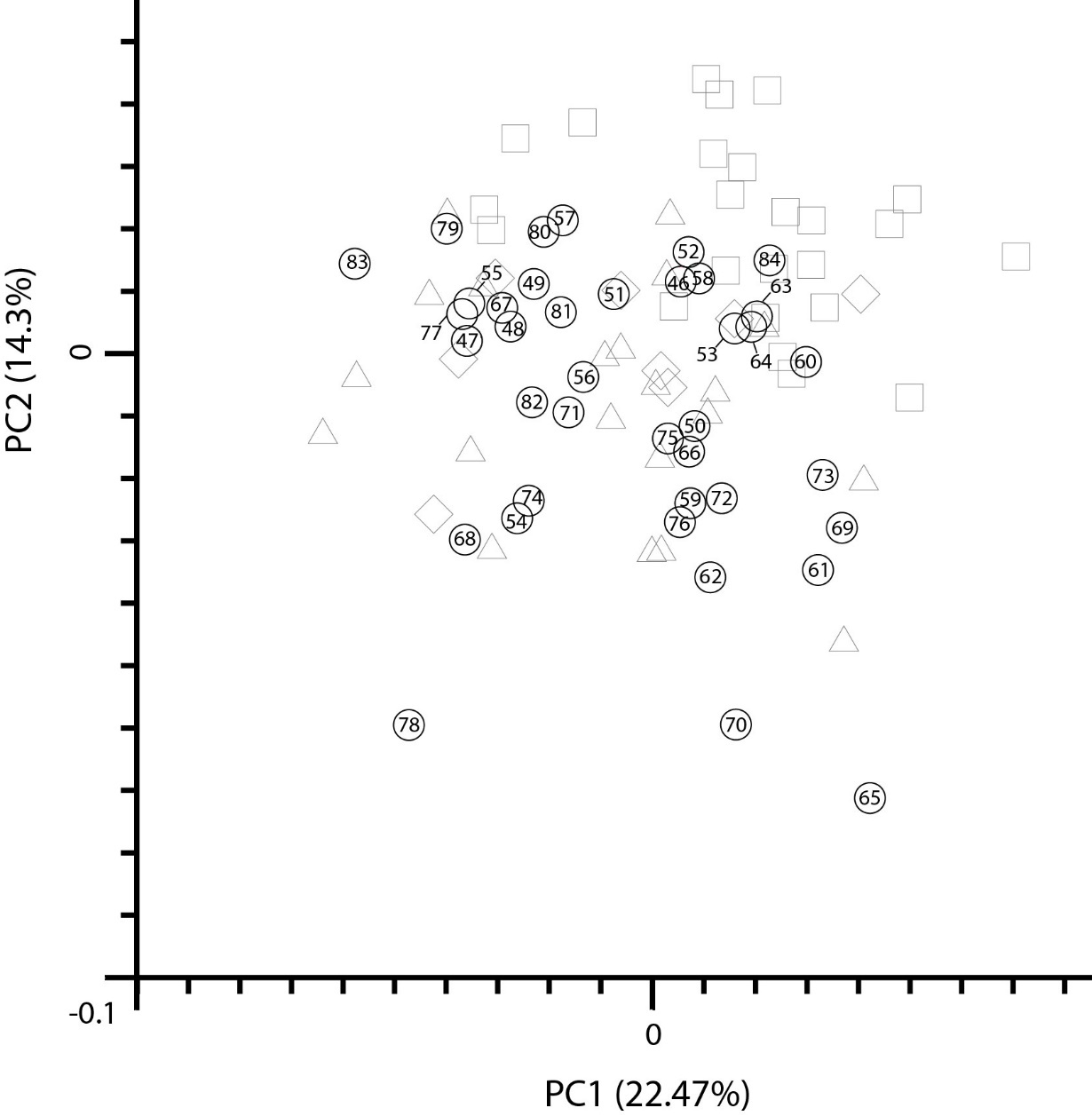


**Figure 2 Percentage of the total variance explained by the first 18 Principal Components**

Variance explained for the first 18 PCs for the principal component analysis of the whole dataset.

**Figure 3 Plot of principal component 2 against principal component 3 on whole dataset**

PC2 (14.3%) against PC3 (10.6%) for the PCA performed on the whole dataset (red squares: wolves; blue triangles: 'prehistoric' specimens; black circles: dogs; green rhombi: dingoes).


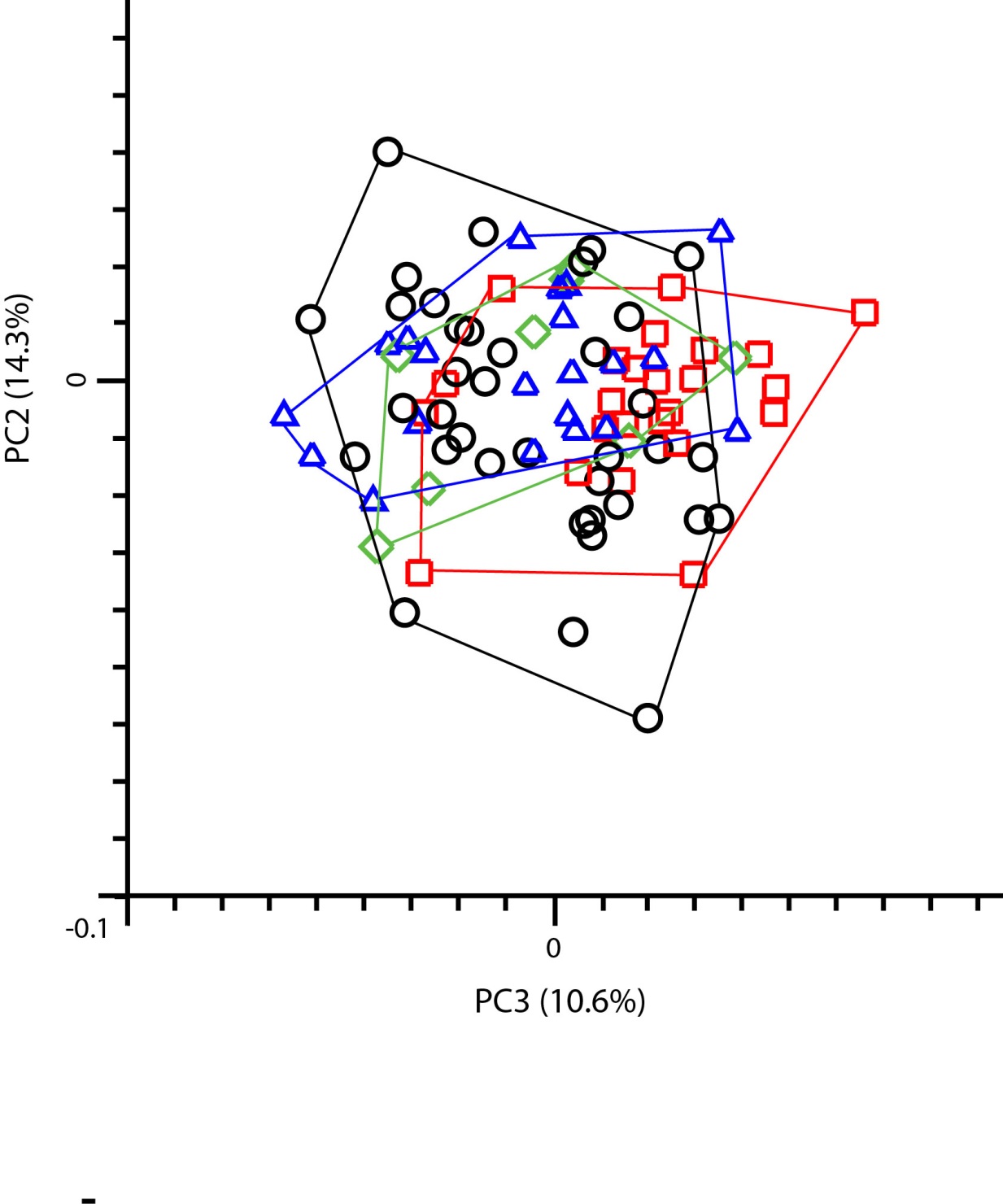


**Figure 4 Principal component analysis on the semicircular canals**

PCA performed on a subset of 68 semilandmarks on the three semicircular canals, plot of PC1 (21.48%) against PC2 (12.25%) (red squares: wolves; blue triangles: 'prehistoric' specimens; black circles: dogs; green rhombi: dingoes).


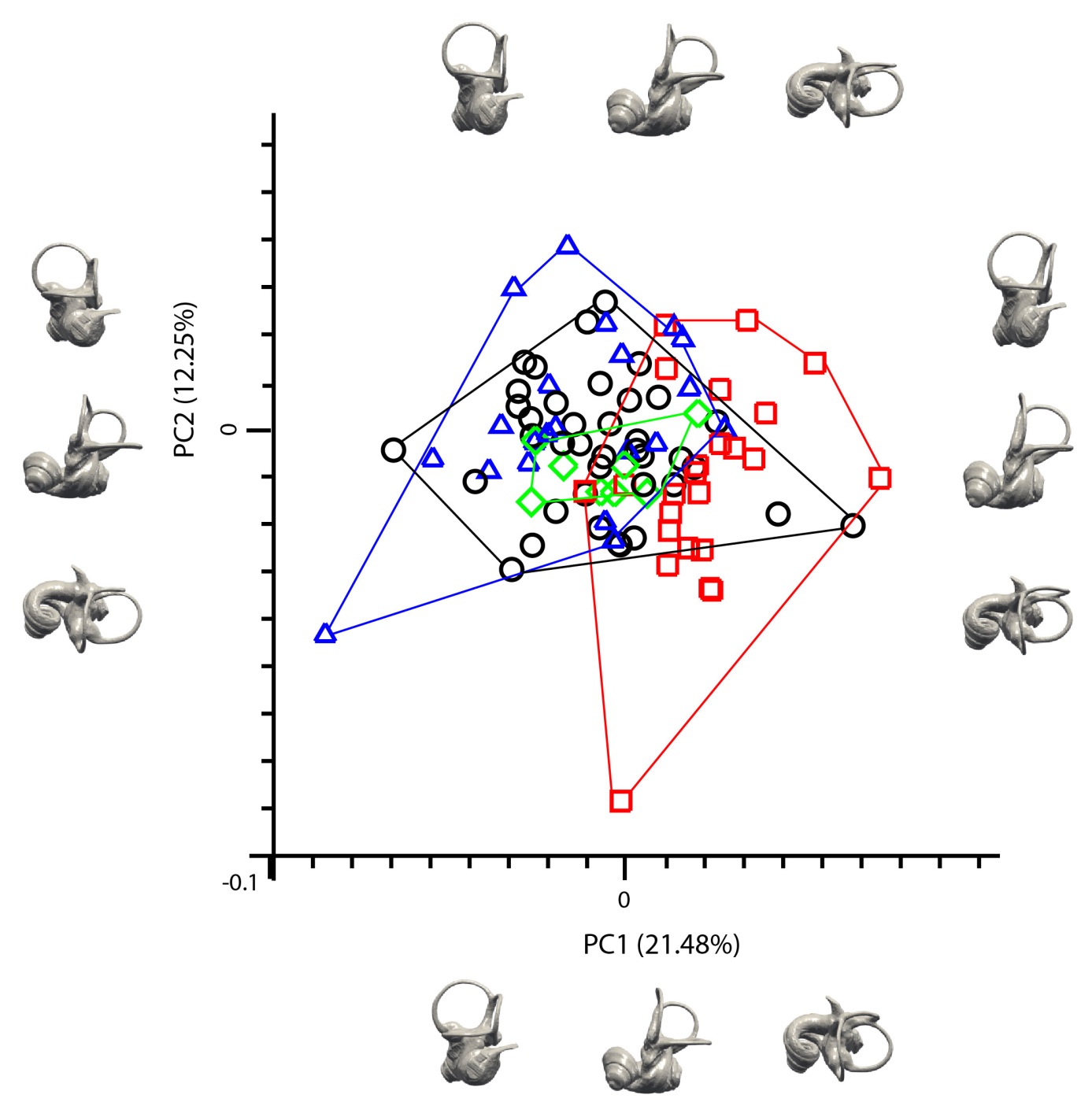

Supplement: Supplementary file 1 — Supplementary 7 tables and 4 figures [file 41598_2017_13523_MOESM1_ESM.docx]
